# Laparoendoscopic Single-Site Surgery (LESS) for Right Donor Nephrectomy in a Patient With Situs Inverses Totalis: A Novel Approach for Such a Case

**DOI:** 10.7759/cureus.55758

**Published:** 2024-03-07

**Authors:** Abdullah M Almalki, Jehad Fikri, Toufik M Jouhar, Ahmed Khalaf, Ghaleb A Aboalsamh

**Affiliations:** 1 Department of Urology, King Faisal Specialist Hospital and Research Centre, Jeddah, SAU; 2 Department of Urology, King Abdullah Medical City, Makkah, SAU; 3 Department of General Surgery, King Faisal Specialist Hospital and Research Centre, Jeddah, SAU; 4 Department of Transplant Surgery, King Faisal Specialist Hospital and Research Centre, Jeddah, SAU

**Keywords:** situs inversus totalis, nephrectomy, kidney transplant, laparoendoscopic single site surgery, laparoscopic donor nephrectomy

## Abstract

Situs inversus totalis (SIT) is a rare congenital condition where the organs of the thorax and abdomen are arranged in a mirror image reversal of their normal position. Patients with SIT present unique challenges in surgical procedures, particularly in laparoscopic surgeries, due to the need to reverse the operator's perspective, technical difficulty in handling the instruments, anatomical variations, and an increased risk of intraoperative complications. In this case report, we present the first case in the English literature of a 49-year-old Arabic male patient with SIT who underwent a successful right laparoendoscopic single-site surgery donor nephrectomy. We described the surgical technique used and highlighted the key challenges faced and overcome during the procedure.

## Introduction

Situs inversus totalis (SIT) is a rare congenital condition where the organs of the thorax and abdomen are arranged in a mirror image reversal of their normal position [1]. This condition affects approximately one in 10,000 individuals worldwide [1]. Patients with SIT present unique challenges in surgical procedures, particularly in laparoscopic surgeries, due to the need to reverse the operator's perspective, technical difficulty in handling the instruments, anatomical variations, and increased risk of intraoperative complications [2].

Robotic, open, and hand-assisted laparoscopic donor nephrectomy have been described more frequently in the literature. However, laparoendoscopic single-site surgery (LESS) for donor nephrectomy has emerged in a few centers worldwide, showing low complication rates and decreased length of stay as well. This well-established approach is expected to be a safe and effective method of performing donor nephrectomy in donors with SIT in experts' hands [3].

In this unique case report, we present the first case in the literature for a patient with SIT who underwent a successful right LESS for donor nephrectomy. We described the surgical technique used and highlighted the key challenges faced and overcome during the procedure.

## Case presentation

**Figure 1:**
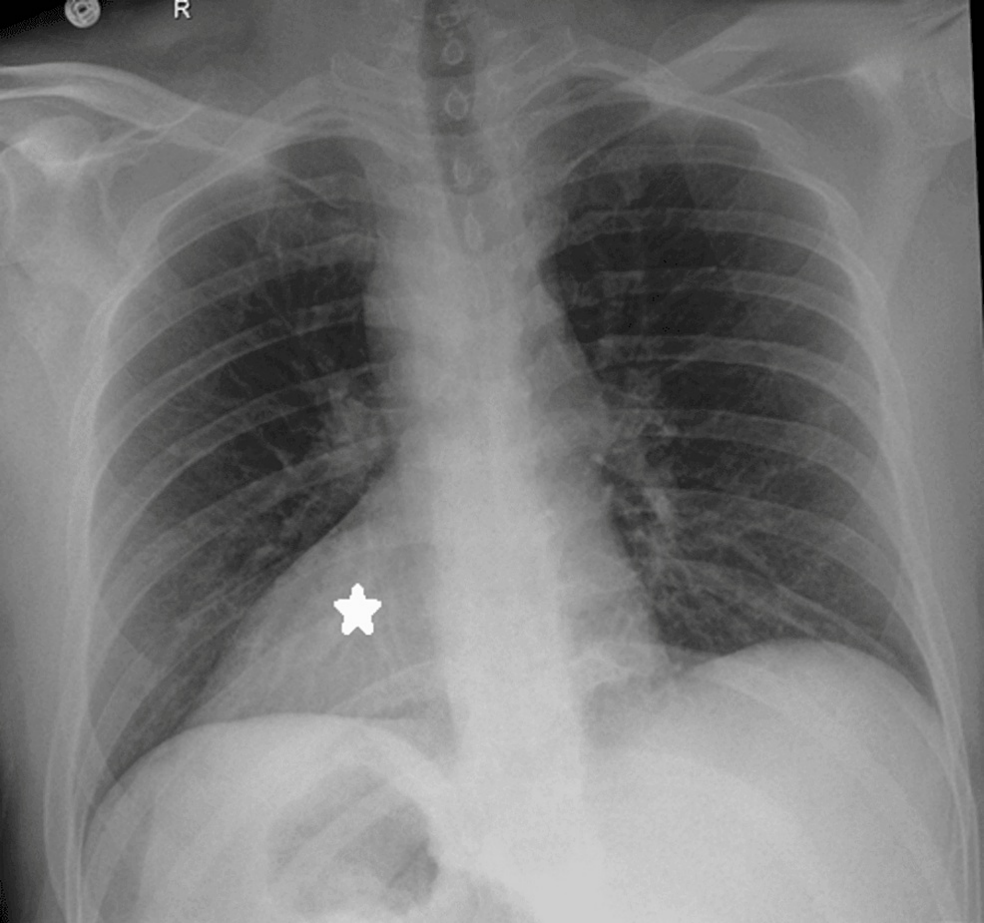
Chest X-ray showing dextrocardia with no signs of pulmonary disease The star denotes the heart in the dextrocardia position.

**Figure 2:**
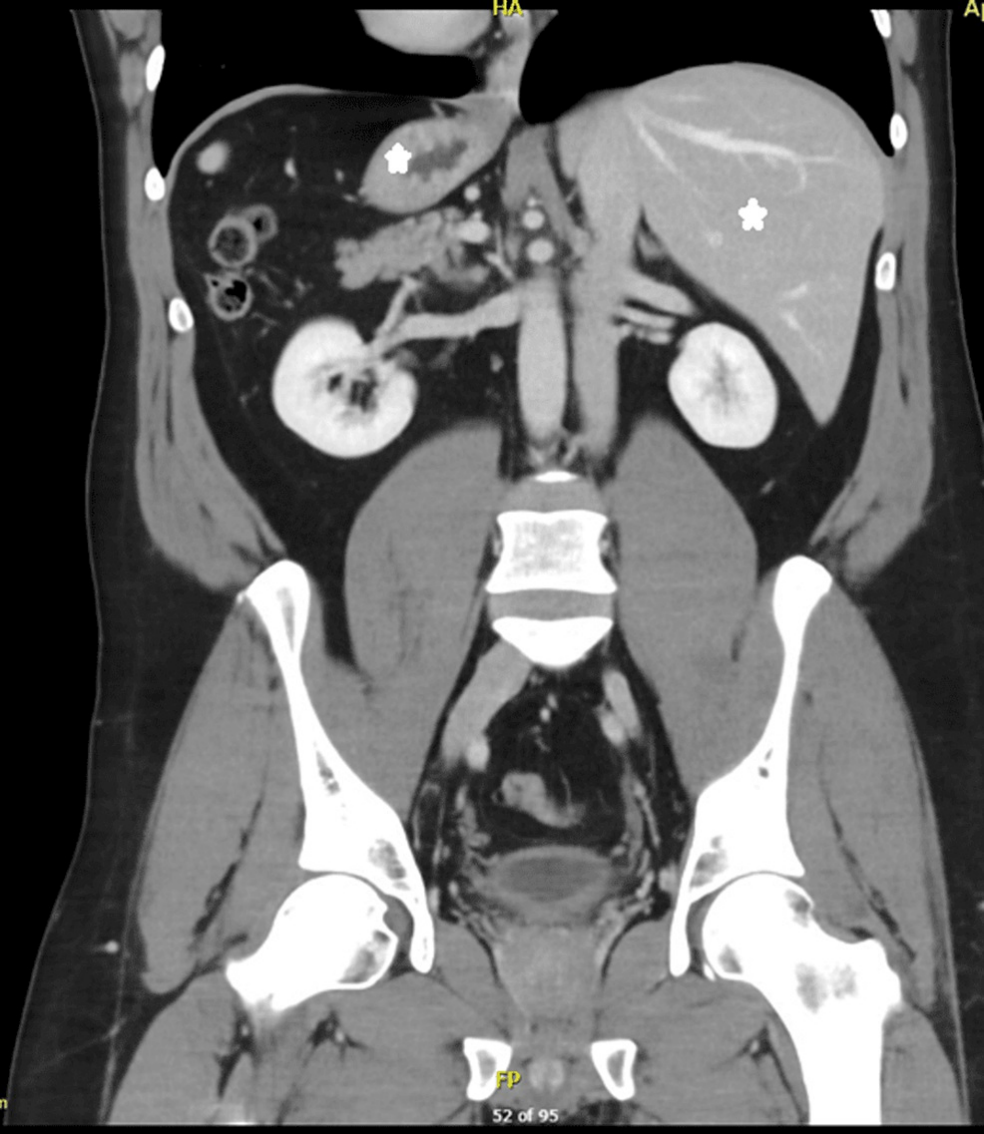
CT of the abdomen with angiogram showing intra-abdominal organs located in reverse, including the liver and stomach, representing SIT The star on the right side denotes the stomach, while the left one denotes the liver. CT: computed tomography; SIT: situs inversus totalis

**Figure 3:**
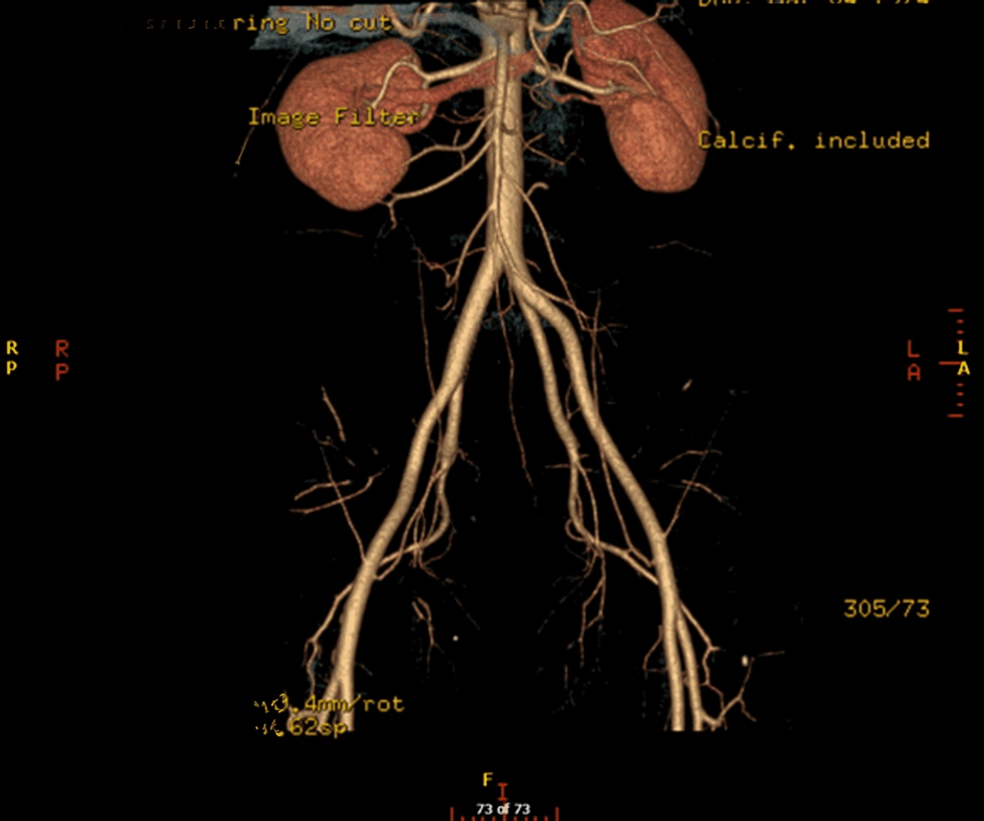
Three-dimensional reconstruction of an abdominal CT with an angiogram showing the kidney’s arterial structure, demonstrating the right and left kidneys with a single artery. The length of the right renal artery from the aorta to the bifurcation is 2.6 cm. The length of the left extrahilar renal artery from the aorta to the bifurcation is 1.8 cm CT: computed tomography

An Arab male patient who is 49 years old with no previous medical or surgical history came for an evaluation to donate his kidney to his sister. The lab results were normal, with a pre-operative creatinine level of 101 µmol/l (normal value 62-106), and a cystatin-based glomerular filtration rate (GFR) of 90 ml/min/1.73m^2^ (normal value 85-155). A chest X-ray revealed dextrocardia (Figure 1). During a CT of the abdomen with an angiogram, it was confirmed that the patient had SIT (Figure 2). A three-dimensional reconstruction of an abdominal CT angiogram shows the kidney’s arterial structure (Figure 3). A nuclear scan showed that both kidneys had a normal cortical function, with a split function of 49% and 51% for the left and right kidneys, respectively, and an average GFR of 101 ml/min. The case was discussed in a multidisciplinary meeting led by the head of transplant surgery and transplant nephrology and their teams, and it was agreed to proceed with the operation. The patient was informed of the risks and benefits at the clinic and gave his written consent.

**Figure 4:**
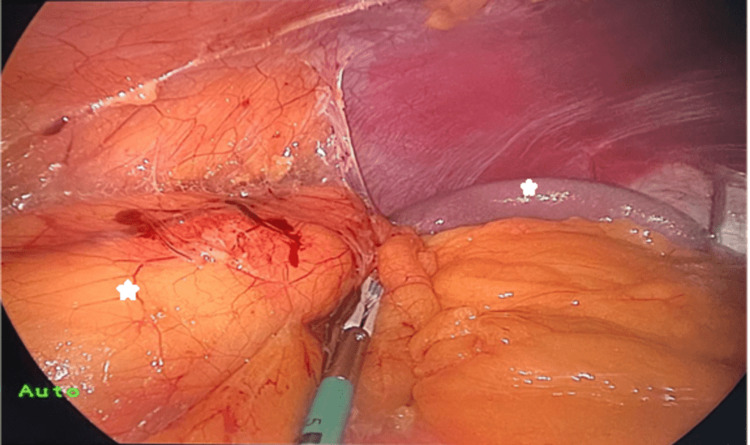
An intraoperative picture showing the spleen and the right kidney on the same side The stars denote the spleen and the right kidney.

As for the surgical approach, the decision was made to take the right kidney; hence, the patient was put in his left lateral decubitus position. Pressure points were padded to prevent compression injuries, then the table was flexed, and the anterior abdomen was prepared and draped in a sterile fashion. The abdominal cavity was opened through a midline periumbilical incision around 5 centimeters along the right curve of the umbilicus, a single GelPOINT platform (Applied Medical Technology, Inc., Ohio, USA) was inserted, and the peritoneal cavity was insufflated [4]. As the abdomen was totally inverted, the descending colon from splenic flexure to sigmoid colon was mobilized using LigaSure (Medtronic plc, Minneapolis, USA) [5]. As shown, the spleen and right kidney were noted (Figure 4). The ureter and gonadal vein were identified at the level of the right common iliac artery and dissected up to the lower pole of the kidney. Gonadal vein traction facilitated renal vein identification and dissection. The adrenal vein was dissected and divided using LigaSure. The renal vein was dissected. The main renal artery was identified and dissected from surrounding tissues. The kidney was mobilized from all around, and the adrenal gland was separated from the upper pole. Following our usual protocol for donors, intravenous furosemide (20 milligrams) was given prior to vascular clamping. The ureter and gonadal vein were clipped and cut. The main renal artery and renal vein were stapled in this order using an ECHELON FLEX Powered Vascular Stapler (Johnson & Johnson Medical Devices, New Jersey, USA) and divided [6]. The kidney was removed in a retrieval Endo bag through the gel point and perfused with the preservation solution. The intraperitoneal cavity, vascular stumps, and staple lines were examined for any bleeding. The midline wound was closed with loop number one polydioxanone suture. The subcutaneous tissue was closed using 3.0 Vicryl, and the skin was covered with a deep dermal monocryl 4.0 stitch. The dressing was applied to the wound. The postoperative course was uneventful, and he was discharged on day one in good condition. Even the clinic visits after that did not reflect any significant surgical complications.

## Discussion

SIT is a rare congenital condition where the organs of the thorax and abdomen are arranged in a mirror image reversal of their normal position [1]. It is also associated with other anomalies such as polysplenia, Ivemark-Kartagener syndrome, and biliary atresia [7-10]. According to Blegen, it was first described by Aristotle in animals, but the writings of Fabricius in 1600 confirmed its existence in humans [11]. Due to its unfamiliar anatomy to the surgeon, any surgery in situs inversus patients faces many difficulties and a higher risk of complications might be anticipated [2]. In the early 20th century, organ donation in situs inversus was contraindicated due to highly prioritizing the donor’s safety [12]. However, situs inversus is no longer a contraindication to donor nephrectomy, with the current advances in surgical techniques. With preoperative planning and a multidisciplinary approach, performing donor nephrectomy in such a population will allow better identification of the anatomical vasculature and potentially decrease the risk of complications.

Our literature review found a total of 10 cases of donor nephrectomy in individuals with SIT [13-20] (Table 1). These cases were reported in eight different studies, with the first one being published by Black et al. in 2003 [13]. The age range of patients who underwent the procedure was between 23 and 70 years old, and most of them were living donors. However, Polak et al. reported a case where the donor was deceased [14]. Laparoscopic surgery was the most commonly used technique, although one case utilized robotic surgery and two cases used an open technique. All cases had successful outcomes, but follow-up periods varied from 30 days to 20 months, with four cases not mentioning follow-up periods. Most of the cases involved retrieving the right kidney, while only two involved taking the left one. This report presents the case of a 49-year-old man who underwent a successful LESS right donor nephrectomy and was followed up in the clinic after 10 days with good health and manageable pain.

**Table 1:** Summary of the total cases reported in the literature regarding donor nephrectomy in SIT since 2003 SIT: situs inversus totalis

Year	Age	Authors	Number of kidneys retrieved	Characteristics	Surgery technique	Outcomes	Follow-up period
2003	27	Black et al. [13]	1	Living donor right kidney	Hand-assisted laparoscopy	Successful	Unknown
2006	23	Polak et al. [14]	2	Cadaveric donors both kidneys	Open nephrectomy	Successful	Unknown
2010	31	Hoffmann et al. [15]	1	Living donor right kidney	Open nephrectomy	Successful	One year
2010	41, 51	van Dellen et al. [16]	2	Living donor right kidney	Hand-assisted laparoscopy	Successful	Unknown
2013	37	Berber et al. [17]	1	Living donor right kidney	Conventional laparoscopic nephrectomy	Successful	Six months
2015	34	Petrović et al. [18]	1	Living donor left kidney	Conventional laparoscopic nephrectomy	Successful	20 months
2016	34	Gonzalez-Heredia et al. [19]	1	Living donor right kidney	Robotic hand-assisted nephrectomy	Successful	Unknown
2020	70	Benjamens et al. [20]	1	Living donor left kidney	Hand-assisted laparoscopy	Successful	30 days
Our case	49	Almalki et al.	1	Living donor right kidney	Laparoendoscopic single-site surgery	Successful	30 days

Various methods for performing donor nephrectomy in SIT have been documented in the literature, including open surgery, conventional laparoscopy, and robotic surgery. However, there was none with the use of LESS, at least in English literature. LESS was initially utilized in gynecologic surgery in 1991 [21]. Moreover, it offers several advantages, such as improved cosmetic outcomes, reduced morbidity from using multiple ports, shorter hospital stays, and the ability to convert to conventional multiple ports with less difficulty than converting to open surgery. Nonetheless, LESS presents challenges such as limited retraction and triangulation due to the restricted space available [22].

## Conclusions

LESS for donor nephrectomy in SIT is a novel approach that can be considered in expert hands. With preoperative planning and a multidisciplinary approach, performing donor nephrectomy in this population will allow better identification of the anatomical vasculature and potentially decrease the risk of complications. Further studies are needed to evaluate the safety and efficacy of this approach in a larger population.
